# Early Years of Carbapenem-Resistant *Enterobacterales* Epidemic in Abu Dhabi

**DOI:** 10.3390/antibiotics11101435

**Published:** 2022-10-19

**Authors:** Tibor Pál, Aqdas B. Butt, Akela Ghazawi, Jens Thomsen, Tahir A. Rizvi, Ágnes Sonnevend

**Affiliations:** 1 Department of Medical Microbiology and Immunology, College of Medicine and Health Sciences, United Arab Emirates University, Al Ain P.O. Box 17666, United Arab Emirates; 2 Department of Medical Microbiology and Immunology, Medical School, University of Pécs, 7624 Pécs, Hungary; 3 Abu Dhabi Public Health Center, Abu Dhabi P.O. Box 5674, United Arab Emirates; 4 Zayed Center for Health Sciences, United Arab Emirates University, Al Ain P.O. Box 17666, United Arab Emirates

**Keywords:** Middle East, carbapenem-resistant *Enterobacterales*, carbapenemase, molecular typing, *Klebsiella pneumoniae* clones

## Abstract

Recent studies showed that the current endemic of carbapenem-resistant *Enterobacterales* (CRE) in the Emirate of Abu Dhabi is dominated by highly resistant *Klebsiella pneumoniae* clones ST14, ST231, and CC147, respectively. In the absence of continuous, molecular typing-based surveillance, it remained unknown whether they lately emerged and rapidly became dominant, or they had been present from the early years of the endemic. Therefore, antibiotic resistance, the presence of carbapenemase and 16S methylase genes, and the sequence types of CRE strains collected between 2009 and 2015 were compared with those collected between 2018 and 2019. It was found that members of these three clones, particularly those of the most prevalent ST14, started dominating already in the very early years of the CRE outbreak. Furthermore, while severely impacting the overall antibiotic resistance patterns, the effect of these clones was not exclusive: for example, increasing trends of colistin or decreasing rates of tigecycline resistance were also observed among nonclonal isolates. The gradually increasing prevalence of few major, currently dominating clones raises the possibility that timely, systematic, molecular typing-based surveillance could have provided tools to public health authorities for an early interference with the escalation of the local CRE epidemic.

## 1. Introduction

Clinical isolates of *Enterobacterales* resistant to carbapenems due to the production of various carbapenemases had already been sporadically encountered during the last decade of the 20th century, but the threat they represent became global toward the end of the first decade of the 21st century [1]. Countries of the Arabian Peninsula have not been spared from this burden, either. Multiple studies of varying scale and details have reported the local emergence of such isolates. With the exception of few [2,3], most of these studies, however, were restricted to the identification of the major groups of carbapenemases produced, and larger studies seldom provided a detailed molecular analysis of the clones present [4,5,6,7,8,9,10,11,12,13,14,15,16,17,18].

In the United Arab Emirates, just as well as in other countries of the region, the methodology needed for such molecular typing has not been broadly applied by public health laboratories for the surveillance of carbapenem-resistant *Enterobacterales* (CRE). Consequently, the emergence and spread of clones of the unfolding CRE epidemic have not been closely followed. Only as recently as in 2018, a relatively small-scale research project revealed in Dubai the dominance of the highly resistant *Klebsiella pneumoniae* sequence type (ST) 14 strains [19]. This was subsequently confirmed by a recent, nationwide study detecting those strains in all major hospitals included in the study across the country [20]. The same study also identified the simultaneous high prevalence of *K. pneumoniae* ST147 and, in two regions, of *K. pneumoniae* ST231 strains.

These prevalence reports, however, did not provide any insight on the timely changes of the local CRE epidemiology, time of emergence, and dynamics of spread of the clones currently dominating the local CRE scene. Answering these questions, even retrospectively, however, could provide helpful information to plan early preventive strategies to mitigate future epidemics and to highlight the importance of early, molecular typing-based, continuous surveillance to interfere even with the initial spread of highly resistant pathogens. In the current, retrospective study, we analyzed CRE strains collected in the hospitals of the Abu Dhabi Emirate in 2009–2015, i.e., during the early years of the local CRE epidemic. The data obtained were compared with those extracted from a recent, nationwide study [20] to reveal if the currently dominating clones had already been present from the beginning, or their emergence and spread were more recent events.

## 2. Results

### 2.1. Characteristics of Collection and Coverage of CRE Studied

Altogether, 394 strains collected between 2009 and 2015 were included in the study. *K. pneumoniae* was the most common species encountered (82.2%) followed by *Escherichia coli* (11.4%), *Enterobacter* sp. (3.8%), and other *Enterobacterales* (2.5%). Urine was the most common sample type (24.2%) followed by respiratory (21.1%), wound and tissue (17.5%), blood (16.6%), and rectal or skin screening specimen (20.6%). The overall coverage rate of CRE encountered in hospitals A–E reporting to Abu Dhabi Antimicrobial Resistance Surveillance Program was 11.7%, exhibiting some variation between hospitals (Table S1) and increasing from 0.93% in 2010 to 21.45% in 2015 (data not shown).

The rates of antibiotic nonsusceptibility are shown in Table S2, and the overall frequency of resistance genes, their alleles, and their presence in different years are presented in Table 1.

The overall high rates of nonsusceptibility to ceftazidime-avibactam, colistin, and tigecycline were particularly noteworthy. Sixty-five percent of the strains exhibited meropenem minimum inhibitory concentration (MIC) values of >8 mg/L, and 11.7% were either extremely or pandrug-resistant (XDR or PDR, respectively). Overall, strains were nonsusceptible to 11.28 ± 2.17 antibiotics of the 17 tested. Eighty-four percent of the strains carried a carbapenemase gene (data not shown). Significantly more carbapenemase producers (67.0%) exhibited meropenem MIC values >8 mg/L than their nonproducing counterparts (6.3%) (*p* < 0.0001). The carriage rate of the *bla*_OXA-48-like_ gene exceeded that of *bla*_NDM_. Nevertheless, the high frequency of the latter one, together with the strains carrying both genes and with a few isolates with bla_VIM_, resulted in a high rate (40.4%) of MBL producers. Nearly 50% of the isolates carried at least one 16S methylase gene (Table 1).

### 2.2. Characterization of Major Clones Present

The 45 *E. coli* strains represented 14 pulsotypes, including 3 singletons (data not shown). Sequence types ST38, ST69, ST156, ST131, ST167, ST410, ST1196, and ST1284 were identified, but none of them qualified by our arbitrary definition (present with >10 members) as a major clone. Representatives of international clones ST38, ST69, and ST131 expressed a variety of carbapenem resistance mechanisms. Two of the ST38 strains produced OXA-48, one expressed NDM-1, the single ST69 isolate carried *bla_OXA-232_*, two ST131 did not produce carbapenemase, and one expressed NDM-1.

The 324 *K. pneumoniae* strains were assigned to 45 pulsotypes (data not shown). The results of the MLST analysis revealed ST14, ST231, clonal complex (CC) 147 (including single-locus variants (SLVs) ST273 and 392), ST11, ST15, ST29, ST45, ST101, ST188, ST307, ST471, ST711, ST2098, and ST2150 correspondingly, of which the first three qualified as major clones. The three major clones together represented 58.6% of the species and almost half (48.2%) of the CRE encountered until 2015. Each of the major clones was present in 5 out of 6 hospitals studied (Table 2).

Members of the major clones were significantly more resistant than their sporadic counterparts as assessed by several resistance-related parameters (Table 3). Although even in ST14 the rate of OXA-48-like carbapenemases exceeded that of NDM, the very high ratio of ST14 strains coproducing these two carbapenemases resulted in a high rate of MBL producers among them with the concomitant high frequency of nonsusceptibility to ceftazidime–avibactam. Moreover, this clone was most commonly resistant to colistin contributing to the high frequency of XDR or PDR strains among them (Table 3).

### 2.3. Changing Rates of Major Clones, Resistance Genes, and Antibiotic Nonsusceptibility over Time

**Figure 1:**
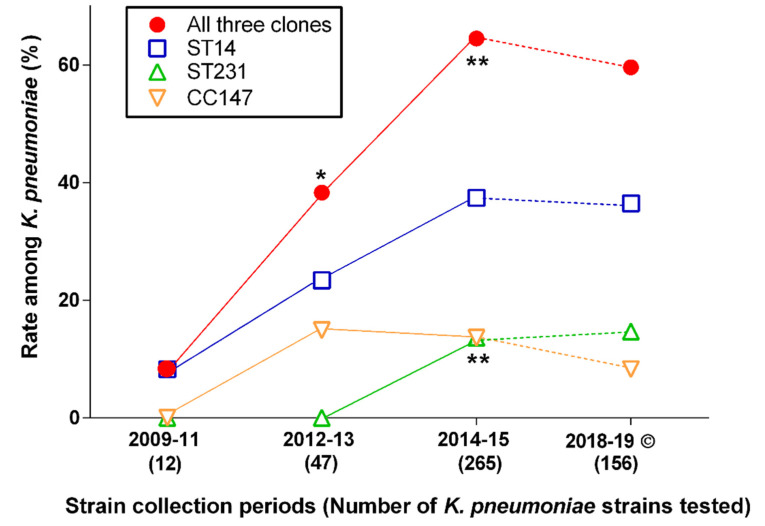
Changing the rates of major *K. pneumoniae* clones over time. Continuous line: current study period; dashed line: changes until the most recent data © [20]. * Significantly (*p* < 0.05); ** highly significantly (*p* < 0.01) different from previous point of time.

Since their first encounter during the first study period, the combined rates of the three major K. pneumoniae clones represented an increasing proportion within the species reaching a peak in 2014–2015. Of them, ST14 strains were the first to be encountered in Abu Dhabi as early as 2011. After that, with the exception of 2012, representatives of this clone have been present all throughout the study period reaching over 35% of *K. pneumoniae* in 2014–2015. CC147 isolates were first isolated in 2012 reaching the highest rate in the same period (i.e., 2012–2013) followed by a moderate decrease thereafter. The latest major clone appearing in Abu Dhabi was the *K. pneumoniae* ST231 in 2014. Importantly, the rate of neither of the three clones has significantly changed after 2014–2015 when compared with more recent data of 2018–2019 [20] (Table 1 and Figure 1).

**Figure 2:**
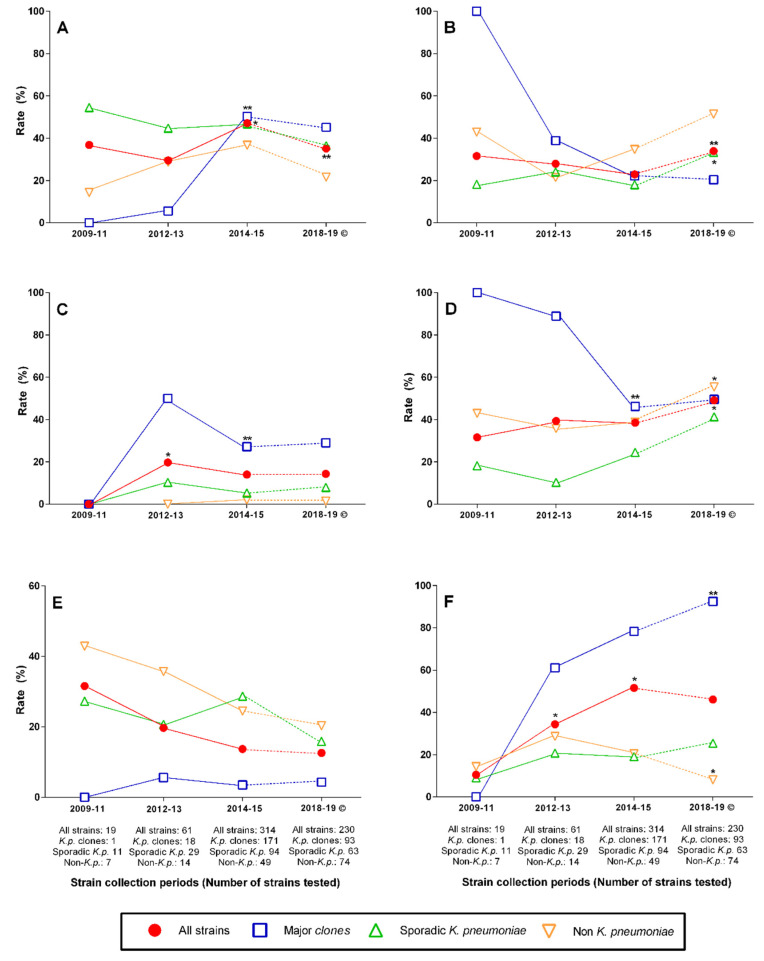
Changing rates of resistance-related genes over time: (**A**) *bla*_OXA-48-like_, (**B**) *bla*_NDM_, and (**C**) *bla*_OXA-48-like_ and *bla*_NDM_. (**D**) Any metallo-beta-lactamase genes. (**E**) No carbapenemase genes. (**F**) Any 16S methylase genes. Continuous line: current study period; dashed line: changes until the most recent data © [20]. * Significantly (*p* < 0.05); ** Highly significantly (*p* < 0.01) different from previous point of time.

All throughout the study period, the rate of *bla*_OXA-48-like_ carrying strains exceeded that of isolates with *bla*_NDM_. However, when all MBL producers were considered (i.e., *bla*_NDM_, *bla*_NDM_-*bla*_OXA-48-like_, and *bla*_VIM_ carriers) with the exception of 2014–2015, i.e., the period with the highest rate of OXA producers, their rate exceeded those of carrying only a class D carbapenemase gene (Figure 2).

After the first collection period (i.e., 2009–2011), double carbapenemase producers have been present at a rate of over 10%. The rate of CRE not carrying any of the five carbapenemase genes tested and not exhibiting any carbapenemase activity sharply dropped after 2012–2013 and has remained low since, below 15%. The opposite trend was seen with 16S methylase genes. Their absence among the few isolates of the first collection period was followed by a sharp increase taking their rates above 50% in 2014–2015 followed by a moderate decrease only, which did not reach the level of statistical significance (Figure 2).

The impact of the major clones on these general trends was the most apparent regarding NDM and OXA-48-like coproducers, whereas a trend among non-*K. pneumoniae* strains affected the most the rate of non-carbapenemase producers, as this has always been rare among members of the major clones. It was noteworthy that while the most considerable increase in the rate of 16S methylases was observed among members of the major *K. pneumoniae* clones, a similar, albeit less pronounced trend was also seen among sporadic strains of the same species (Figure 2).

**Figure 3:**
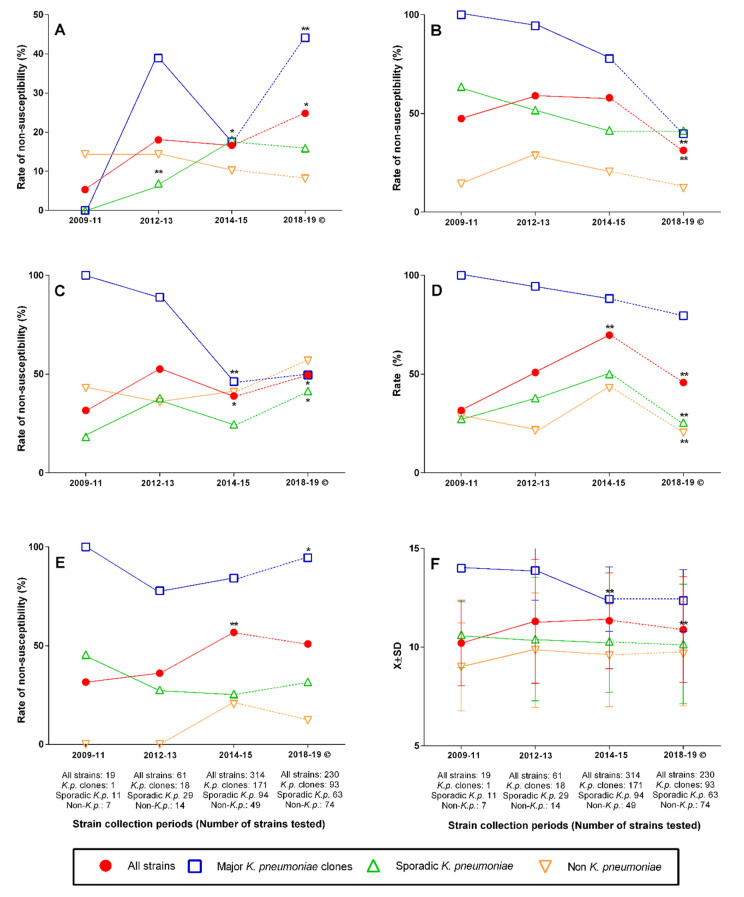
Changing rates of selected antibiotic resistance parameters over time. (**A**) Colistin, (**B**) tigecycline, (**C**) ceftazidime–avibactam, (**D**) meropenem MIC > 8 mg/L, (**E**) amikacin, and (**F**) R index. Continuous line: current study period; dashed line: changes until the most recent data © [20]. * Significantly (*p* < 0.05); ** Highly significantly (*p* < 0.01) different from the previous point of time.

While the overall nonsusceptibility to tigecycline exhibited a relatively stable level among all strains but major *K. pneumoniae* clones, the rate of nonsusceptibility among the members of the latter group continuously declined all throughout the study period and continued thereafter. In sharp contrast to this, the overall nonsusceptibility to colistin increased, with a significant further elevation until recently. This was mostly driven, particularly after 2014–2015, by the members of the major *K. pneumoniae* clones (Figure 3).

The trends of nonsusceptibility to ceftazidime–avibactam mirrored those of MBL producers (Figure 2 and Figure 3). Except for members of the major clones, among which high-level meropenem resistance was almost a rule with a slight, nonsignificant decrease over time, sporadic *K. pneumoniae* and non-*K. pneumoniae* strains increased their resistance level to meropenem until 2014–2015, which was followed by a considerable, significant decline. The overall R index, except for a significant decline of the respective values of the major clones after 2012–2013, has been relatively stable being the highest among major clones and the lowest among non-*K. pneumoniae* isolates (Figure 3).

## 3. Discussion

In countries of the Arabian Peninsula, and specifically in the UAE, most publications on CRE either reported the first encounters with specific carbapenemase producing *Enterobacterales* or provided the prevalence of CRE expressing various carbapenemases in a region or in specific hospitals within a specific, usually short, timeframe [4,5,6,7,8,9,10,11,12,13,14,15,16,17,18]. Systematic, continuous surveillance studies relying on strain typing have not addressed the unfolding of the CRE outbreak. Therefore, whether the features of the current CRE epidemic, i.e., dominance of a few *K. pneumoniae* clones (ST14, CC147, and ST231), and a high rate of double carbapenemase producers and MBLs [19,20,21] are a relatively recent phenomenon or have developed much earlier and have been maintained since remained unknown.

The current study clearly showed that the ratios of these three clones, albeit at different speed, increased over time practically from the very early years of the outbreak, and by as early as 2014–2015, their combined rate exceeded 60% with a slight, nonsignificant decline subsequently. This increasing trend was the most pronounced regarding ST14 isolates (Figure 1), i.e., the clone was still the most prevalent type in 2018–2019 [20]. Furthermore, ST14 strains encountered between 2011 and 2015 were the most resistant (Table 3), i.e., a feature continuously observed in the more recent strain collection [20]. It should be noted that, in the latter study, ST14 strains were significantly more commonly isolated from Emirati than from non-Emirati patients [20]. Regretfully, whether this association had already been there from the beginning could not be addressed in the current study, as no data of the patients’ nationality for these early strains were available to us.

An important observation was that, although, due to their high proportion, major clones considerably impacted several parameters of the entire pool of strains studied, their effect was not exclusive. In some cases, comparable tendencies were observed among sporadic *K. pneumoniae* and among other species as well (e.g., the rates of *bla*_OXA-48-like_ carriers, double carbapenemase producers, and regarding sporadic *K. pneumoniae* carriers of any 16S methylase genes) (Figure 2). The decline in the rate of noncarbapenemase producers was largely determined by sporadic *K. pneumoniae* and non-*K. pneumoniae* strains, as this feature comparatively remained rare among members of the major *K. pneumoniae* clones (Figure 2). Considering the extremely high ratios of MBL producers among members of the major clones (in this case, only the ST14 and CC147 clones (Table 3)) during the first two collection periods, care should be exercised as the numbers of strains were still low at those times. However, it is noteworthy that the frequency of MBL expressing sporadic isolates and non-*Klebsiella* strains continuously increased until the most recent times, and, hence, all groups contributed to the current high rates of MBL producers and, consequently, to the high rate of ceftazidime–avibactam resistance (Table 2 and Table 3).

Despite some variations in the rates of strains producing different carbapenemase (Figure 2) all throughout the study until the most recent data [20], OXA- and NDM-type enzyme-producing strains have been the most common ones encountered. This is in contrast with observations at other parts of the world, where in hospitals or in entire countries, noticeable changes have taken place over the years in the types of enzymes produced by CRE [22,23,24]. In some hospitals, this was attributed to the increasing use of therapies ineffective against MBL producers leading to the selection of strains expressing class B type enzymes [22]. On the other hand, some studies reported the increase in KPC-type enzyme producers over time, gradually exceeding isolates expressing other carbapenemases, either locally, in a hospital [23], or in larger regions [24]. The first KPC-producing strain was first isolated in the UAE, in Dubai [12], and since then, they have been only occasionally encountered [20]. The reason for the comparative local absence of this type of strains is unknown, just as well whether their paucity could have contributed to the apparently stable dominance of OXA and NDM producers.

Investigating the presence of various aminoglycoside-modifying enzymes has been out of the frames of the current study. Nevertheless, the role of 16S methylases in the overall aminoglycoside resistance of local CRE is suggested by the fact that, after 2014–2015, amikacin nonsusceptibility has moderately declined only among non-*Klebsiella* CRE, whereas it has increased among clonal and sporadic *K. pneumoniae* strains mirroring the trends regarding the presence of 16S methylase genes among them (Figure 2 and Figure 3).

The increasing rate of colistin resistance of the local CRE was mostly determined by the increasing proportion of members of the major clones, although a slight increase among sporadic *K. pneumoniae* was also simultaneously present with a moderate decrease among non-*Klebsiella* isolates (Figure 3). It should be noted that during the study period (i.e., 2009–2015), in the absence of clear technical guidelines, several laboratories either used the unreliable disk diffusion or automated susceptibility testing methods or did not test for colistin susceptibility, likely contributing to the excessive use of this drug. Although the presence of transferable colistin resistance genes was not studied in the current project, previous observations suggest that, while common among animal isolates [25], plasmid-mediated colistin resistance is either absent or relatively rare among clinical isolates in the region [19,20].

In the absence of antibiotic prescription data, we can only speculate that the decline in the rates of tigecycline nonsusceptibility in all groups of isolates, particularly after 2012–2013, could be due to improved stewardship practices (Figure 3). On the other hand, based on the timely changes of the types of carbapenemases produced, we cannot explain the pattern seen regarding the rate of strains with meropenem MIC > 8 mg/L, i.e., a slight but continuous decrease among members of the major clones contrasted by an increase among other strains until 2014–2015 followed by a sharp decline. Once again, in the absence of the respective clinical data, the role of more accurate meropenem dosing in the very recent years remains speculative.

A considerable weakness of the current study is that exposing strains to more detailed WGS analyses was beyond its limits. Hence, we do not know whether the heterogeneity recently observed among ST14-type strains [26] has already been there at the initial expansion of this sequence type. However, recent studies indicated both considerable differences, and in a few cases, remarkable similarities among ST14 strains were encountered in different hospitals [20]. This suggests that beyond local emergence or import, interhospital transfers could play a significant role in the development of the current scenario.

## 4. Materials and Methods

**Strain collection**. Between 2009 and 2015, our laboratory at the College of Medicine and Health Sciences (CMHS), UAE University, offered a service to detect the genes of the five most common carbapenemases in CRE strains isolated at Abu Dhabi hospitals. Laboratories could submit nonrepeat *Enterobacterales* isolates exhibiting nonsusceptibility to any of the carbapenems by any of the methods used. Strains were obtained from 5 major government hospitals (Hospitals A–E), managed by the Abu Dhabi Health Service Company (SEHA), and also from another nonprivate hospital (Hospital F) (Table S1).

Although, in 2013, the Health Authority of Abu Dhabi (currently Department of Health, Abu Dhabi) issued a circular advising laboratories to use this service [27], hospitals have not been under any obligation to comply, and strain submission had been voluntary during the entire study period.

Strains were included in the study irrespective of whether they were isolated from clinical or patients’ screening samples but not from environmental specimens. No samples were collected for the sake of the study, all patient identifiers were masked, and we did not have access to databases containing patients’ data. To estimate the coverage of CRE encountered in Abu Dhabi, the number of isolates studied was compared with that of nonrepeat CRE isolates reported to the Abu Dhabi Antimicrobial Resistance Surveillance Program (AD ARS) covering all but one hospital (Hospital F) since 2010 (Jens Thomsen–unpublished).

When analyzing changes in time, the period between 2010 and 2015 was divided into a single 3-year-long and two 2-year-long periods (2009–2011 and 2012–2013 and 2014–2015, respectively). These retrospective data were also compared with more recent (2018–2019) figures from Abu Dhabi extracted from the first nationwide CRE surveillance study [20]

Strains were stored at −80 °C in Tryptic Soy Broth (TSB, MAST, Bootle, UK) containing 10% glycerol.

**Antibiotic susceptibility testing.** The methods to determine quantitative susceptibilities to ceftazidime, ceftazidime–avibactam, cefotaxime, imipenem, meropenem, ertapenem, aztreonam, aztreonam–avibactam, trimethoprim–sulfamethoxazole, ciprofloxacin, gentamicin, amikacin, tetracycline, chloramphenicol, fosfomycin, colistin, and tigecycline, as well as the interpretation and criteria labeling of strains as pan- or extremely drug-resistant (PDR and XDR, respectively), were previously described [16]. A “resistance index” was calculated indicating the number of antibiotics out of the 17 tested to which an isolate exhibited nonsusceptibility. In case of meropenem, strains exhibiting MIC > 8 mg/L were distinguished. The production of carbapenemases was assessed by the CIM test [28].

**Detection of the antibiotic resistance genes.** The detection of *bla*_NDM_, *bla*_OXA-48-like_, *bla*_VIM_, *bla*_IMP_, and *bla*_KPC_; determination of the alleles of *bla*_NDM_, *bla*_OXA-48-like_, and *bla*_VIM_; and detection of 16S methylase genes *armA, rmtA, rmtB, rmtC*, and *rmtD* were carried out as previously described [14]. Carriages of *rmtE* and *rmtF* were detected according to Davis et al. [29] and Hidalgo et al. [30], respectively.

**Molecular typing of the isolates**. *XbaI* digested genomes were separated by pulsed field gel electrophoresis (PFGE) as described [14,31]. *E. coli* and *K. pneumoniae* clones with high prevalence were identified by the following arbitrary criteria: PFGE clusters exhibiting ≥80% pattern similarities and containing more than 4 members were further divided into subclusters exhibiting ≥90% pattern similarities. In each subcluster, representatives of each unique carbapenem resistance mechanisms (i.e., carrying specific carbapenemase genes, the combination of, or none of them) were randomly selected and subjected to MLST analysis [32,33]. A cluster of strains exhibiting ≥80% PFGE similarity to strains with a specific sequence type (ST) or clonal complex (CC) including single-locus variants of the respective ST thus identified were considered to represent a major clone if they have more than 10 members. Strains not belonging to major clones thus identified were considered sporadic.

**Sequencing.** PCR amplicons were sequenced for allele determination and for MLST using the Big Dye Cycle Terminator V.3.1 on a 3130X analyzer (Applied Biosystems, Badford, MA, USA).

**Statistical analysis.** The level of association of features with groups of strains was assessed by the chi-squared test, whereas differences in particular rates of features were analyzed by Student’s unpaired *t*-test (GraphPad Prism v6.07, GraphPad Software Inc., San Diego, CA, USA).

## 5. Conclusions

As the data presented in this study are not based on case-control observations, any possible effect of a timely, systemic, molecular typing-based surveillance of MDR strains on the increasing trends of the local CRE prevalence remains speculative. Nevertheless, the current study showed that members of the major clones currently dominating in the region had already been present, albeit undetected, during the early years of the CRE epidemic. This raises the possibility that timely investigations leading to the early identification of their sources and common routes of their transmission could have a mitigating effect on the escalation of the outbreak. In our views, such real-time surveillance work could also be highly beneficial in the future to alleviate the nationwide spread of other emerging multiresistant pathogens. In countries where public health laboratory systems with the proper methodological capacities are still rudimentary or nonexistent, their establishment could considerably improve surveillance including that of MDR pathogens.

## Figures and Tables

**Table 1:** Frequency and yearly presence of resistance genes, their alleles, clones, and certain resistance-related characteristics.

Characteristics	Genes, Clones or Resistance Features	% within the Collection	Alleles	% within the Enzyme Group	Year Encountered						
					2009	2010	2011	2012	2013	2014	2015
Resistance genes	*bla_NDM_*	24.9	*bla_NDM-1_*	89.8	+	+	+	+	+	+	+
			*bla_NDM-4_*	1.0						+	
			*bla_NDM-5_*	8.2						+	+
			*bla_NDM-7_*	1.0				+			
	*bla_OXA-48-like_*	43.9	*bla_OXA-48_*	37.0	+	+	+	+	+	+	+
			*bla_OXA-162_*	1.7						+	+
			*bla_OXA-181_*	16.8					+	+	+
			*bla_OXA-232_*	42.8					+	+	+
			*bla_OXA-244_*	1.7					+	+	+
	*bla_VIM_*	0.8	*bla_VIM-4_*	66.6				+	+		
			*bla_VIM-55_*	33.3							+
	*bla_NDM_* + *bla_OXA-48-like_*	14.5	*bla_NDM-1_* + *bla_OXA-48_*	8.9					+		+
			*bla_NDM-1_* + *bla_OXA-162_*	1.8				+			
			*bla_NDM-1_* + *bla_OXA-181_*	3.6						+	+
			*bla_NDM-1_* + *bla_OXA-232_*	73.2					+	+	+
			*bla_NDM-5_* + *bla_OXA-181_*	12.5					+	+	+
	*bla_NDM-1_* + *bla_VIM-55_*	0.3	*bla_NDM-1_* + *bla_VIM-55_*	100.0							+
	Any MBL ^1^	40.4	-	-	+	+	+	+	+	+	+
	Any 16S methylase	49.6	*armA*	30.5			+	+	+	+	+
			*rmtB*	1.3					+	+	+
			*rmtC*	1.3					+		+
			*rmtF*	15.7				+	+	+	+
Resistance-related characteristics	XDR	9.1	-	-		+	+	+	+	+	+
	PDR	2.5	-	-				+	+	+	+
	Colistin R	16.2	-	-			+	+	+	+	+
	Tigecycline R	57.6	-	-	+	+	+	+	+	+	+
	Ceftazidime–avibactam R	40.6	-	-	+	+	+	+	+	+	+
*K. pneumoniae* major clones	ST14	28.2	-	-			+		+	+	+
	CC147 ^2^	10.9	-	-				+	+	+	+
	ST231	11.1	-	-						+	+

^1^ MBL—metallo beta-lactamase, ^2^ CC—clonal complex, including single-locus variants (SLVs) ST147, ST273, and ST392.

**Table 2:** Rate and distribution of major *Klebsiella pneumoniae* clones encountered between 2009 and 2015.

Group	*N*	Rate (%) among		Presence in Hospitals ^3^
		*K. pneumoniae*	All Strains	
ST14	111	34.3	28.2	A, B, C, D, F
CC147 ^1^	43	13.3	10.9	A, B, C, D, F
ST231	36	11.1	9.1	A, B, C, D, E
All major clones	190	58.6	48.2	A, B, C, D, E, F
Sporadic ^2^ *K. pneumoniae*	134	41.4	34.0	A, B, C, D, E, F

^1^ Including single-locus variants (SLVs) ST147, ST273, and ST392. ^2^ All strains not belonging to any major clones were considered sporadic. ^3^ Hospital codes see in Table S1).

**Table 3:** Selected features of major *Klebsiella pneumoniae* clones encountered between 2009 and 2015 as compared with sporadic isolates.

Groups	N	Rate (%) of												
		Resistance Genes							Nonsusceptibility to			Resistance-Related Parameters		
		*bla_NDM_*	*bla_OXA-48-like_*	*bla_NDM_* + *bla_OXA-48-like_*	MBL ^3^	Any 16S Methylase	*armA*	*rmtF*	Ceftazidime-Avibactam	Colistin	Tigecycline	Meropenem MIC > 8 mg/L	XDR or PDR	R Index
ST14	111	30.3	36.9	**32.4**	**63.1**	**78.4**	**78.4**	0	**67.6**	**26.1**	**75.7**	**89.2**	**33.3**	**13.02 ± 1.29**
ST231	36	**0.0**	**97.2**	0.0	**0.0**	**100.0**	5.6	**97.2**	**0.0**	5.6	**100.0**	**91.7**	0.0	**12.27 ± 0.77**
CC147 ^1^	43	34.9	37.2	14.0	**48.8**	**51.2**	11.6	**39.5**	**48.8**	14.0	**72.1**	**86.1**	9.3	**11.67 ± 2.51**
Sporadic ^2^ strains	134	12.8	30.1	3.9	17.2	12.2	7.8	2.5	17.7	9.3	30.0	30.0	2.0	10.35 ± 2.63

^1^ Including single-locus variants (SLVs) ST147, ST273, and ST392. ^2^ All strains not belonging to any major clones were considered sporadic. ^3^ MBL—metallo beta-lactamase. Gray shadow: the value is significantly (*p* < 0.05) different from that of sporadic strains. Gray shadow with bolded characters: the value is highly significantly (*p* < 0.01) different from that of sporadic strains.

## Data Availability

Not applicable.

## Supplementary Materials

The following supporting information can be downloaded at: https://www.mdpi.com/article/10.3390/antibiotics11101435/s1, Table S1: Characteristics of hospitals providing samples for the study; Table S2: Rate of antibiotic nonsusceptibility, MIC50, and MIC90 values of 394 CRE isolated between 2009 and 2015.
