# Supplementary material for: Early Years of Carbapenem-Resistant Enterobacterales Epidemic in Abu Dhabi
Source: Antibiotics (Basel). 2022 Oct 19;11(10):1435. doi: 10.3390/antibiotics11101435 (PMC9598120; doi:10.3390/antibiotics11101435)
Supplement: Supplementary file 1 [file antibiotics-11-01435-s001.zip › antibiotics-1936664-supplementary.pdf]

Table S1. Characteristics of hospitals providing samples for the study

Hospitals	Type	Size (beds) *	Contribution to the study (%)	Overall coverage rate in the hospital during the study period (%)**
A	Tertiary care	450	31.2	14.8
B	Tertiary care	550	26.9	10.3
C	Tertiary care	450	23.9	12.6
D	Acute care and emergency	400	12.4	12.4
E	Secondary care	200	2.5	4.0
F	Tertiary care	350	3.0	No data

**approximate figures **based on figures from 2010 onwards, counting not-repeat isolates, only*

Table S2. Rate of antibiotic non-susceptibility, MIC50 and MIC90 values of the 394 CRE isolated between 2009 and 2015

Antibiotics	%	MIC50	MIC90
		(mg/L)	
Ceftazidime	93.9	>128	>128
Ceftazidime/Avibactam	40.6	2	>128
Cefotaxime	95.9	>128	>128
Ertapenem	100.0	>64	>64
Imipenem	89.1	16	128
Meropenem	84.5	16	64
Aztreonam	89.9	>128	>128
Aztreonam/Avibactam	4.3	0.5	1
Ciprofloxacin	92.1	64	>64
Gentamicin	74.6	128	>256
Amikacin	52.3	32	>256
Trimethoprim-Sulfamethoxazole	85.0	>256	>256
Tetracycline	56.6	8	>256
Chloramphenicol	75.3	64	>256
Colistin	16.2	<0.5	16
Tigecycline	57.6	2	4
Fosfomycin	21.3	16	64
